# Macrophage Plasticity and Polarization Are Altered in the Experimental Model of Multiple Sclerosis

**DOI:** 10.3390/biom11060837

**Published:** 2021-06-04

**Authors:** Alessandro Leuti, Emanuela Talamonti, Antonietta Gentile, Marta Tiberi, Alessandro Matteocci, Diego Fresegna, Diego Centonze, Valerio Chiurchiù

**Affiliations:** 1 Department of Medicine, Campus Bio-Medico University of Rome, 00128 Rome, Italy; a.leuti@unicampus.it; 2 Laboratory of Neurochemistry of Lipids, European Center for Brain Research (CERC)/IRCCS Santa Lucia Foundation, 00143 Rome, Italy; 3 Department of Molecular Biosciences, The Wenner-Gren Institute, University of Stockholm, 114 Stockholm, Sweden; emanuela.talamonti@dbb.su.se; 4 Synaptic Immunopathology Lab, IRCCS San Raffaele Pisana, 00163 Rome, Italy; gntnnt01@uniroma2.it (A.G.); diego.fresegna@gmail.com (D.F.); 5 Laboratory of Resolution of Neuroinflammation, European Center for Brain Research (CERC)/IRCCS Santa Lucia Foundation, 00143 Rome, Italy; m.tiberi@hsantalucia.it (M.T.); m.matteocci@hsantalucia.it (A.M.); 6 Department of Systems Medicine, Tor Vergata University, 00133 Rome, Italy; centonze@uniroma2.it; 7 Unit of Neurology, IRCCS Neuromed, 86077 Pozzilli, Italy; 8 Institute of Translational Pharmacology, National Research Council, 00133 Rome, Italy

**Keywords:** multiple sclerosis, EAE, macrophages, cytokines, chemokines, toll-like receptors

## Abstract

Multiple sclerosis (MS) is an immune-mediated demyelinating disease of the central nervous system. MS is characterized by infiltrations of leukocytes such as T and B lymphocytes and macrophages. Macrophages have been identified as major effectors of inflammation and demyelination in both MS and its animal model, experimental autoimmune encephalomyelitis (EAE). However, the activation and heterogeneity of macrophages in MS has been poorly investigated. Thus, in this study, we evaluated M1 and M2 macrophages immunophenotype from EAE and control mice by analyzing over 30 surface and intracellular markers through polychromatic flow cytometry, qRT-PCR, and ELISA assay. We showed that M1 macrophages possessed a higher proinflammatory profile in EAE compared to control mice, since they expressed higher levels of activation/co-stimulatory markers (iNOS, CD40, and CD80) and cytokines/chemokines (IL-6, IL-12, CCL2, and CXCL10), whereas M2 lost their M2-like phenotype by showing a decreased expression of their signature markers CD206 and CCL22, as well as a concomitant upregulation of several M1 makers. Furthermore, immunization of M1 and M2 macrophages with MOG35-55 led to a significant hyperactivation of M1 and a concomitant shift of anti-inflammatory M2 to pro-inflammatory M1 macrophages. Overall, we provide evidence for a phenotypic alteration of M1/M2 balance during MS, which can be of crucial importance not only for a better understanding of the immunopathology of this neurodegenerative disease but also to potentially develop new macrophage-centered therapeutic strategies.

## 1. Introduction

Multiple sclerosis (MS) is a chronic inflammatory demyelinating disease of the central nervous system (CNS) that remains a major cause of disability. Several studies demonstrated that MS lesions contain multiple leukocyte cell types including lymphocytes, macrophages, and dendritic cells, all of which are believed to contribute to lesion formation by various distinct and interacting mechanisms [1,2,3]. Among these leukocyte subsets, infiltrating macrophages have been identified as major effectors of inflammation and demyelination in both MS and experimental autoimmune encephalomyelitis (EAE), the animal model of MS [4,5]. These findings have stimulated intense interest on the various effector functions of macrophages in lesion formations. Macrophages exhibit considerable heterogeneity with respect to their receptor expression and pathways leading to several degrees of activation and polarization in response to the inflammatory cytokine milieu and/or environmental cues (e.g., microbial products, damaged cells, activated lymphocytes). Mirroring the Th1/Th2 nomenclature, many refer to polarized macrophages (Mϕ) such as M1-Mϕ and M2-Mϕ, which represent extremes of a continuum in a universe of activation states, where classically activated M1-Mϕ produce many proinflammatory and effector molecules. M2-Mϕ is a generic name for various forms of activated macrophages involved in protective and immunoregulatory functions [6,7,8,9]. Indeed, CNS white matter lesions are particularly enriched in activated microglial cells as well as infiltrating macrophages [10] that are able to produce high amounts of reactive oxygen and nitrogen species (ROS and RNS), leading to the massive axonal and oligodendrocyte damage associated with MS clinical course [11]. Furthermore, infiltration of highly activated macrophages in the CNS can be observed during the induction and symptomatic stages of EAE, acting as major effectors of the disease [12] and correlating with disease severity. Conversely, resolution and remission of the symptoms are characterized by fewer numbers of infiltrated macrophages [13], suggesting that macrophages derived from peripheral monocytes are key players in MS onset and progression. Even though there is much evidence that suggests an alteration of the M1/M2 balance in MS pathogenesis [14], the impact of M1 and M2 macrophages in EAE is still elusive, mainly due to the use of very few markers for these cell populations and a lack of a complete immunophenotypical profile.

On the basis of this scenario, the aim of the present work was to thoroughly investigate the immunophenotype and functional profile of the different macrophages’ subtypes in EAE in terms of their different activation status and plasticity through an ex vivo large-scale characterization of surface activation/co-stimulatory markers, cytokines, chemokines and chemokine receptors, as well as toll-like receptors (TLR).

## 2. Materials and Methods

### 2.1. Animals

C57BL/6 mice (Charles-River, Sulzfeld, Italy) were randomly assigned to standard cages (4–5 animals per cage) and kept at standard housing conditions with a light/dark cycle of 12 h and free access to food and water. Beginning one week before the immunization, all animals were kindly handled every day to reduce the stress induced by operator manipulation during behavioral experiments. Animal experiments were performed according to the Internal Institutional Review Committee, the European Directive 2010/63/EU and the European Recommendations 526/2007, and the Italian D.Lgs 26/2014.

### 2.2. EAE Induction and Clinical Evaluation

EAE was induced in 7–8-week-old female C57BL/6 animals (n = 10), as previously described [15,16]. Briefly, control animals (n = 10) received the same treatment as EAE mice without the immunogen MOG peptide (CFA). The animals were scored daily for clinical symptoms of EAE, according to the following scale: 0 = healthy; 1 = flaccid tail; 2 = ataxia and/or paresis of hindlimbs; 3 = paralysis of hindlimbs and/or paresis of forelimbs; 4 = tetraparalysis; 5 = moribund or death due to EAE. Intermediate clinical signs were scored adding 0.5 value. Moreover, CFA mice daily manipulated daily to avoid the influence of different handling on behavioral outcomes among experimental groups. In EAE mice, first clinical symptoms appear about 10–12 days post immunization (dpi) with a peak of severity at about 19–21 dpi. This stage is referred to as symptomatic or acute phase of the disease. Spleens and cells were extracted during this stage.

### 2.3. M1 and M2 Macrophages Polarization

Spleens from CFA and EAE C57BL/6 mice were obtained and they reduced to a single cell suspension with a 70 μm cell strainer in order to separate connective tissue from splenocytes. Splenocytes were depleted of CD3^+^ T cells using MACS beads. The resulting CD3^−^ negative splenocytes (i.e., monocyte-enriched cells, 0.5 × 10^6^ cells per well) were left in adhesion for 2 h in RPMI 1640 supplemented with 10% FCS, 1% L-glutamin, 1% Na-pyruvate, and 1% penicillin/streptomycin in FCS-coated dishes at a density of (measure) cells/cm^2^ in order for monocytes to adhere. All medium reagents were purchased from Lonza (Basel, Switzerland). After 2 h, non-adherent cells were removed and adhering monocytes were gently rinsed with PBS and cultured in fresh complete medium supplemented with 50 ng/mL of M-CSF for 6 days. At days 2 and 4, cells were provided with new medium supplemented with 25 ng/mL of M-CSF. At day 6, cells were rinsed with PBS and polarized into M1 in presence of 100 ng/mL LPS and 10 ng/mL mouse IFN-γ or into M2 in presence of 20 ng/mL IL-4 for 2 more days [17]. All mouse recombinant cytokines were purchased from Miltenyi Biotec (Bergisch Gladbach, Germany). Supernatants were then collected and stored at −20 °C until further assays were performed to assess cytokine release. Adherent M2 or M2 macrophages were collected and analyzed for immunophenotyping or molecular biology techniques. In some experiments, 6-day-old M1 and M2 macrophages were immunized with 20 μg/mL MOG together with their specific polarizing factors (LPS + IFN and IL-4, respectively) for 2 days.

### 2.4. Flow Cytometry

For surface staining of activation markers, M1-Mϕ and M2-Mϕ stained with several conjugated-antibodies (see Table 1) for 10 min at 4 °C, as reported by [17]. For intracellular staining of iNOS and CD68, M1-Mϕ and M2-Mϕ were fixed with 4% formaldehyde for 10 min at RT, and then stained intracellularly with anti-iNOS primary antibody in 0.5% saponine, at RT for 30 min, washed with PBS, and then stained with Alexa488-conjugated anti-mouse secondary antibody (1:100) for 30 min. The expressions of surface markers and intracellular iNOS were analyzed via flow cytometry in a FACS-Cyan ADP (Beckman Coulter™, Pasadena, CA, USA) by gating them on CD68 + F4/80+ macrophages. For each analysis, at least 10,000 events were acquired.

### 2.5. RNA Extraction and Gene Expression Analysis by Real-Time PCR

Total RNA was extracted using Reliaprep™ extraction columns (Promega, Madison, USA), analyzed at Nanodrop 2000c (Thermo Fisher Scientific, Waltham, MA, USA) in order to assess acid nucleic quantity and quality (using 260/280 nm and 260/230 nm ratios) and then retrotranscripted to obtain cDNA via a Superscript^®^ Vilo™ cDNA synthesis kit (Thermo Fisher Scientific, Waltham, MA, USA), according to the manufacturer’s instructions. Transcripts were quantified using real-time quantitative PCR on an ABI PRISM 7900 sequence detector (Applied Biosystems, Foster City, CA, USA) with an Applied Biosystems predesigned TaqMan Gene Expression Assays and Absolute QPCR ROX mix (Thermo Fisher Scientific, Waltham, MA, USA). The following probes were used (Applied Biosystems, assay identification numbers in parentheses): TLR1 (Mm01208874_m1), TLR2 (Mm00442346_m1), TLR3 (Mm01207404_m1), TLR4 (Mm00445273_m1), TLR5 (Mm00546288_s1), TLR6 (Mm02529782_s1), TLR7 (Mm00446590_m1), TLR8 (Mm04209873_m1), and TLR9 (Mm00446193_m1). In each sample, mRNA quantity was normalized to the amounts of β2-microglobulin (Mm00437762_m1) mRNA. Relative mRNA quantities were expressed as fold over β2-microglobulin mRNA level.

### 2.6. Analysis of Cytokine Release by Luminex Multiplex Assay

Cytokine and chemokine release were tested by performing a custom-made magnetic Luminex^®^ screening assay (R&D) on supernatants obtained by M1 or M2-polarized macrophages obtained from CFA and EAE mice spleens. Each supernatant was accordingly diluted in order to obtain the same volume amount per number of cells (100 µL of cell supernatant/1 × 10^5^ cells) and amounts of cytokines (pg/mL) were measured on a Luminex × 200 (Thermo Fisher Scientific, Waltham, MA, USA).

### 2.7. Statistics

All data were expressed as means ± SEM. Differences between groups were compared using the Student’s *t *test. All statistical analyses were performed with Prism 5.0 (GraphPad Software, San Diego, CA, USA) and *p* values < 0.05 were considered significant. Flow cytometry analysis was performed using the FlowJo analysis program (Treestar, Ashland, OR, USA).

## 3. Results

### 3.1. M1-M2 Immunophenotype in EAE

**Figure 1:**
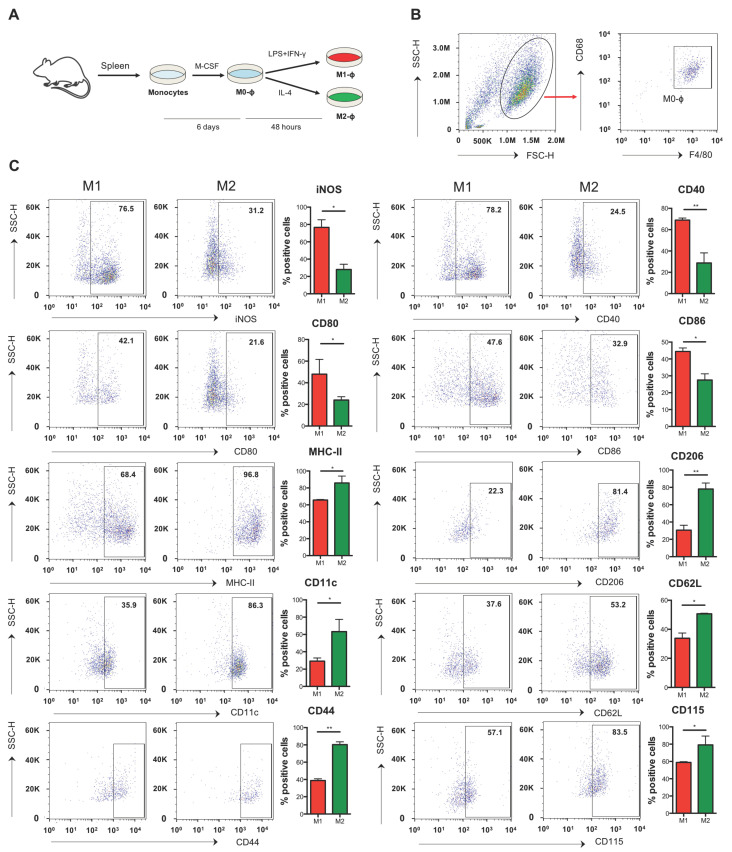
**Immunophenotype of M1 and M2 macrophages obtained from CFA mice.** (**A**) Monocytes obtained from spleen of CFA mice were differentiated into macrophages using M-CSF (50 ng/mL) for 6 days and then polarized into M1 with IFN-γ (10 ng/mL) plus LPS (1 μg/mL) or into M2 with IL-4 (20 ng/mL) for two more days. (**B**) Representative flow cytometry plot showing the gating strategy to identify monocyte-derived macrophages (**C**) Expression of markers by flow cytometry upon staining cells at cell surface (CD40, CD11b, MHC-II, CD80, CD86, CD25, CD206, CD11c, CD62L, CD44, CD115, and CCR5) or intracellularly (iNOS). Data are reported as percentage of positive cells and are representative of 10 independent experiments ± SEM * *p* < 0.05; ** *p* < 0.01.

**Figure 2:**
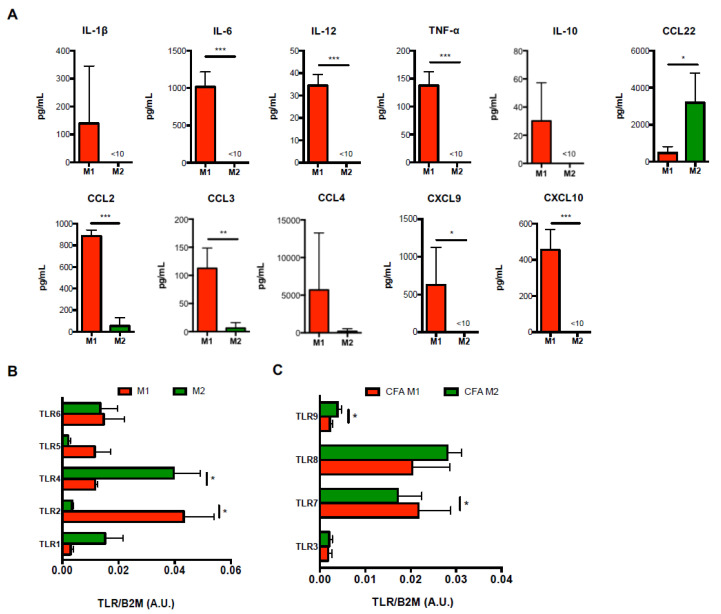
**Expression of cytokines/chemokines and TLRs in M1 and M2 macrophages obtained from CFA mice.** (**A**) ELISA of cytokines (IL-1β, IL-6, IL-12, TNF-α, IL-10) and chemokines (CCL2, CCL3, CCL4, CCL22, CXCL9, and CXCL10) in supernatants of M1 or M2 macrophages. Data are reported as pg/mL and represent 10 independent experiments means ± SEM. * *p* < 0.05, ** *p* < 0.01, *** *p* < 0.001. (**B**,**C**) qRT-PCR analysis of the expression of both bacterial (TLR1, TLR2, TLR4, TLR5, TLR6) (**B**) and viral (TLR3, TLR7, TLR8, TLR9) (**C**) Toll-like receptors in M1 and M2 macrophages. Cycling threshold values are normalized to those of mRNA encoding β2-microglobulin and data are normalized to the maximum value obtained for each donor. Data are expressed as arbitrary units (A.U.) and are shown as means ± SEM of eight independent experiments each in duplicate. * *p* < 0.05.

Since M1-Mϕ and M2-Mϕ nomenclature represent an over-simplistic definition that is intended to describe the extremes of a rather continuous spectrum of macrophage heterogeneity [9], we first conducted an ex vivo large scale characterization of a panel of several known or unprecedently investigated M1/M2 markers including activation markers (iNOS, MHC-II, CD11b, CD11c, CD16, CD25, CD40, CD44, CD62L, CD80, CD86, CD115, CD206), cytokines (IL-1β, IL-6, IL-12, TNF-α, IL-10), as well as chemokines and chemokine receptors (CCL2, CCL3, CCL4, CCL22, CXCL9, CXCL10, CCR5). This multiparametric analysis allowed us to determine a detailed immunophenotypic characterization of murine splenic monocyte-derived and M-CSF-differentiated macrophages (Mϕ) that for simplicity will be referred to as M1-Mϕ and M2-Mϕ, respectively (Figure 1A), and identified within the CD68 + F4/80 population (Figure 1B). In particular, such investigation proved that iNOS, CD40, and CD80/86 (Figure 1C), as well as IL-6, IL-12, TNF-α, CCL2, CCL3, CXCL9, and CXCL10 (Figure 2A), were bona fide markers of M1 polarization and CD206, CD11c, CD44, CD62L, CD115 (Figure 1B), and CCL22 of M2 polarization (Figure 2A) in mice. Interestingly, in our polarization setting, although highly expressed in both M1-Mϕ and M2-Mϕ, MHC-II resulted in significantly higher M2-Mϕ (Figure 1B). Other markers or cytokines/chemokines analyzed from the panel, i.e., CD11b, CD25, and CCR5 (Supplementary Figure S1A), as well as IL-1β, IL-10, and CCL4, did not show any significant differences between M1-Mϕ and M2-Mϕ in order to be labelled as signature markers. Moreover, in order to further characterize their immunophenotype, the expression pattern of toll-like receptors (TLR) was investigated for the first time in M1-Mϕ and M2-Mϕ, evaluating the mRNA levels of the 5 bacterial TLRs (TLR1, 2, 4, 5, 6) and 4 viral TLRs (TLR3, 7, 8, 9). As shown in Figure 2B, among bacterial TLRs, M1-Mϕ displayed higher levels of TLR2 and TLR5 compared to M2-Mϕ, with the former being significantly upregulated, whereas TLR1 and TLR4 were higher in M2-Mϕ, with the latter showing a significant variation. No variation in TLR6 mRNA levels was observed between M1-Mϕ and M2-Mϕ. Furthermore, investigation of viral TLRs denoted a significantly higher expression of TLR8 and partly of TLR9 in M2-Mϕ compared to M1-Mϕ and a significantly higher expression of TLR7 in M1-Mϕ (Figure 2C). The expression levels of TLR3 mRNA were comparable and unchanged between M1-Mϕ and M2-Mϕ. Interestingly, the overall levels of viral TLR3 and TLR9 in both M1-Mϕ and M2-Mϕ were much lower compared to TLR7 and TLR8. The mRNA expression of the various TLRs could be ranked as follows: TLR2>TLR7/8>TLR4/5/6>TLR1>TLR3/9 in M1-Mϕ and TRL4>TLR8>TLR1/6/7>TLR2/9>TLR3/5 in M2-Mϕ.

### 3.2. M1-Mϕ Are Immunophenotypically Hyperactive in EAE

**Figure 3:**
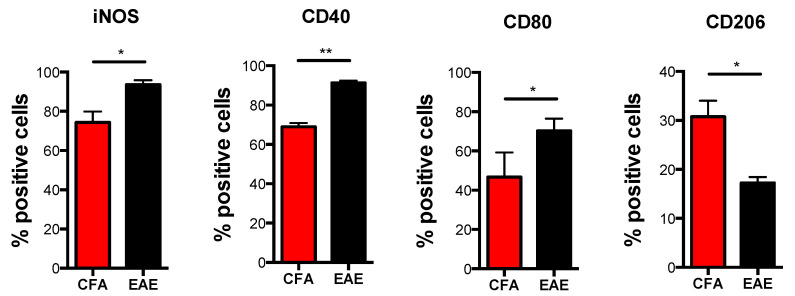
**Immunophenotype of M1 macrophages obtained from CFA and EAE mice.** Expression of markers by flow cytometry upon staining cells at cell surface (CD40, CD80 and CD206) or intracellularly (iNOS) in M1 and M2 macrophages obtained from CFA and EAE mice. Data are reported as percentage of positive cells and are representative of eight independent experiments ± SEM * *p* < 0.05, ** *p* < 0.01.

**Figure 4:**
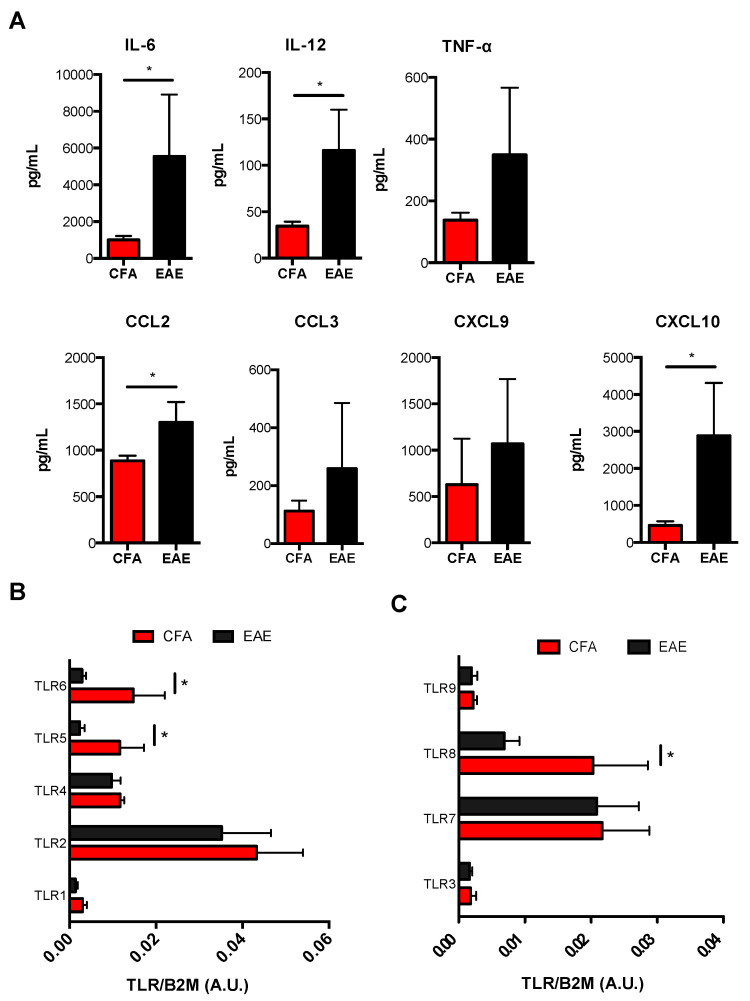
**Expression of cytokines/chemokines and TLRs in M1 obtained from CFA and EAE mice.** (**A**) ELISA of cytokines (IL-1β, IL-6, IL-12, TNF-α, IL-10) and chemokines (CCL2, CCL3, CXCL9, and CXCL10) in supernatants of M1 macrophages. Data are reported as pg/mL and represent 10 independent experiments means ± SEM. * *p* < 0.05 (**B**,**C**) qRT-PCR analysis of the expression of both bacterial (TLR1, TLR2, TLR4, TLR5, TLR6) (**B**) and viral (TLR3, TLR7, TLR8, TLR9) (**C**) Toll-like receptors in M1 macrophages. Cycling threshold values are normalized to those of mRNA encoding β2-microglobulin, and data are normalized to the maximum value obtained for each donor. Data are expressed as arbitrary units (A.U.) and are shown as means ± SEM of eight independent experiments each in duplicate. * *p* < 0.05.

In order to investigate whether M1-Mϕ and M2-Mϕ plasticity and polarization are altered during MS, we analyzed their immunophenotypic and functional profile in EAE mice compared to control mice (CFA) by evaluating those markers that showed significant variations between the two cell types. During EAE, M1-Mϕ undergo a considerable upregulation of their signature markers, including iNOS, activatory molecules CD40 and CD80 (Figure 3), as well as generally higher levels of proinflammatory cytokines IL-6, IL-12, and TNF-α. Chemokines CCL2, CCL3, CXCL9, and CXCL10, with IL-6, IL-12, CCL2, and CXCL10 were shown to be significant (Figure 4A). Furthermore, when checking M2 markers on M1-Mϕ from EAE mice, we noticed that a few of them (i.e., CD206, CD11c and CD115) showed an even lower expression compared to CFA mice, with CD206 being significantly reduced (Figure 3). Other markers were comparable between EAE and CFA mice (Supplementary Figure S1B). In order to verify whether the enhanced pathogenic potential of M1-Mϕ in EAE was paralleled by an altered TLR expression, we measured TLR gene expression in M1-Mϕ obtained from EAE and CFA mice. Figure 4C shows that viral TLRs did not undergo any major variation in their expression during EAE, with TLR8 being the only viral TLR to show a significant reduction, whereas bacterial TLRs displayed the greatest variations. All of them, however, generally decreased in M1-Mϕ from EAE mice. There was a significant reduction for TLR5 and 6 (Figure 4B). Altogether, these results suggest that during EAE there is a global hyperactivation of M1-Mϕ macrophages who produce higher levels of proinflammatory mediators and show a potentially altered TLR-dependent response to pathogens.

### 3.3. M2-Mϕ Lose Their Profile and Switch to M1-Mϕ in EAE

**Figure 5:**
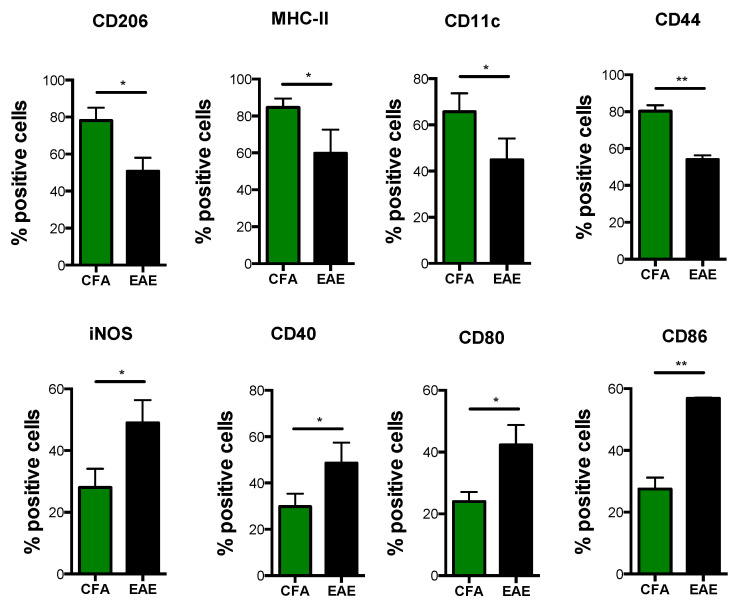
**Immunophenotype of M2 macrophages obtained from CFA and EAE mice.** Monocytes obtained from spleen of CFA and EAE mice were differentiated into macrophages using M-CSF (50 ng/mL) for 6 days and then polarized into M2 with IL-4 (20 ng/mL) for 2 more days. Expression of markers by flow cytometry upon staining cells at cell surface (CD40, MHC-II, CD80, CD86, CD206, CD11c, and CD44) or intracellularly (iNOS). Data are reported as percentage of positive cells and are representative of eight independent experiments ± SEM. * *p* < 0.05, ** *p* < 0.01.

**Figure 6:**
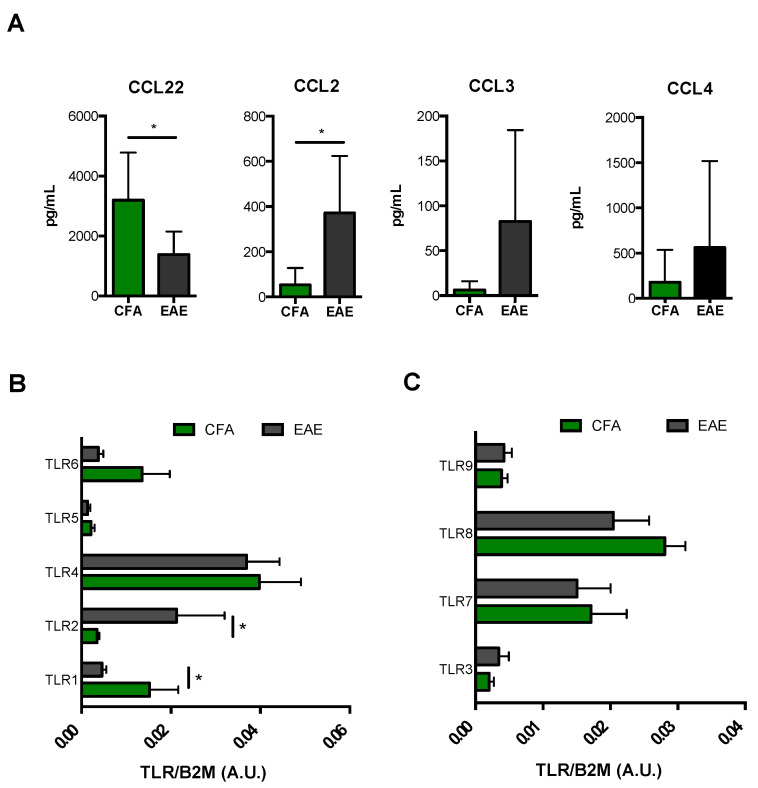
**Expression of cytokines/chemokines and TLRs in M2 obtained from CFA and EAE mice.** (**A**) ELISA of chemokines (CCL2, CCL3, CCL4, and CCL22) in supernatants of M2 macrophages. Data are reported as pg/mL and represent eight independent experiments means ± SEM. * *p* < 0.05 (**B**,**C**) qRT-PCR analysis of the expression of both bacterial (TLR1, TLR2, TLR4, TLR5, TLR6) (**B**) and viral (TLR3, TLR7, TLR8, TLR9) (**C**) Toll-like receptors in M2 macrophages. Cycling threshold values are normalized to those of mRNA encoding β2-microglobulin, and data are normalized to the maximum value obtained for each donor. Data are expressed as arbitrary units (A.U.) and are shown as means ± SEM of eight independent experiments each in duplicate. * *p* < 0.05.

Similar to what we observed in M1-Mϕ, M2-Mϕ displayed a profoundly subverted immunophenotype during EAE. In particular, signature M2 surface markers such as CD206, MHC-II, CD11c, and CD44 were significantly reduced in M2-Mϕ obtained from EAE mice (Figure 5). This was also true for a typical M2 chemokine CCL22 (Figure 6A). Interestingly, only CCR5 and CD115 remained unchanged (Supplementary Figure S1C). Furthermore, the mRNA expression profile of TLRs in M2-Mϕ revealed a substantial rearrangement of such receptors following induction of EAE, particularly bacterial ones. Indeed, EAE mice showed a downregulation of TLR1 and TLR6, with TLR1 being significant, paralleled by a two-fold increase in TLR2 expression. No variation of TLR5 was observed (Figure 6B). On the other hand, EAE poorly influenced the expression pattern of viral TLRs in M2-Mϕ, in that it only elicited a slight insignificant downregulation of TLR8 while not perturbing the transcription of other viral TLRs (Figure 6C). Interestingly, when looking at M1 markers in M2-Mϕ, we observed that EAE-derived M2-Mϕ showed a significant upregulation of several M1 markers, including iNOS, CD40, and CD80/CD86 (Figure 5), as well as CCL2 and to a lesser extent CCL3 and CCL4 (Figure 6A). This suggests that they lost their typical alternatively-activated profile and acquired an immunophenotypical profile similar to M1-Mϕ during EAE M2-Mϕ.

### 3.4. MOG35-55 Alters M1-Mϕ and M2-Mϕ Polarization

**Figure 7:**
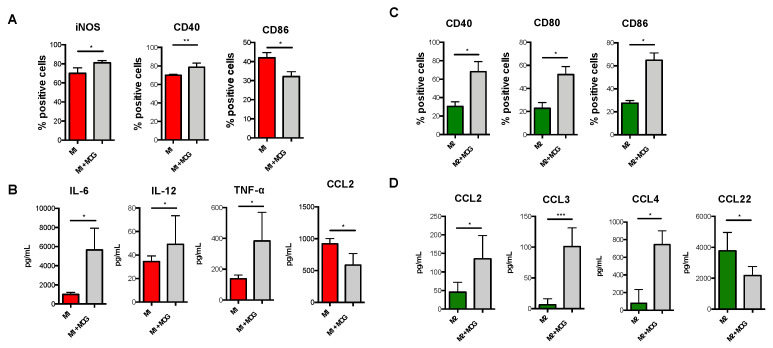
**Immunophenotype of MOG-immunized M1 and M2 macrophages obtained from CFA mice.** Monocytes obtained from spleen of CFA mice were differentiated into macrophages using M-CSF (50 ng/mL) for 6 days and then polarized into M1 with IFN-γ (10 ng/mL) plus LPS (1 μg/mL) or into M2 with IL-4 (20 ng/mL) for 2 more days in the presence of myelin oligodendrocyte glycoprotein epitope (MOG35-55). (**A**) Expression of markers by flow cytometry upon staining cells at cell surface (CD40 and CD86) or intracellularly (iNOS). Data are reported as percentage of positive cells and are representative of four independent experiments ± SEM. * *p* < 0.05, ** *p* < 0.01 (**B**) ELISA of cytokines and chemokines (IL-6, IL-12, TNF-α and CCL2) in supernatants of MOG-immunized M1 macrophages obtained from CFA mice. Data are reported as pg/mL and represent eight independent experiments means ± SEM. * *p* < 0.05 (**C**) Expression of markers by flow cytometry upon staining cells at cell surface (CD40, CD80 and CD86). Data are reported as percentage of positive cells and are representative of eight independent experiments ± sem. * *p* < 0.05, (**D**) ELISA of chemokines (CCL2, CCL3, CCL4, and CCL22) in supernatants of M2 macrophages. Data are reported as pg/mL and represent eight independent experiments means ± SEM. * *p* < 0.05, *** *p* < 0.001.

Additionally, since the autoreactive properties of macrophages in EAE are triggered by myelin recognition, we also asked whether polarization of M1-Mϕ and M2-Mϕ was influenced by immunization with myelin oligodendrocyte glycoprotein-(MOG)-35-55. In order to do so, we treated monocyte-derived macrophages obtained from control mice in the absence or presence of MOG35-55 during their polarization into either M1-Mϕ or M2-Mϕ. Our results showed that MOG35-55-treated M1-Mϕ displayed significantly higher expression levels of iNOS and CD40 and significantly lower levels of CD86 (Figure 7A). Since M1 macrophages released higher levels of pro-inflammatory cytokines and chemokines during EAE, we also verified their release upon treatment with MOG35-55, finding that MOG35-55-treated M1-Mϕ released significantly higher levels of IL-6, IL-12, TNF-α, and CXCL10, but reduced levels of CCL2 (Figure 7B). Other activation markers (CD80) or inflammatory mediators (i.e., IL-1β, CCL3, CCL4, and CXCL9) did not show any significant variation upon MOG35-55 treatment (Supplementary Figure S2A). Conversely, although MOG35-55-treated M2-Mϕ showed no variation of their signature markers CD206, MHC-II, CD11c, and CD44 (Supplementary Figure S2B), they significantly increased the expression of several M1 markers such as CD40, CD80, and CD86, but not of iNOS (Figure 7C and Supplementary Figure S2B), as well as displaying a concomitant and significant higher release of CCL2, CCL3, and CCL4 coupled to a decrease of the M2 chemokine CCL22 (Figure 7D). Other cytokines and chemokines were not detected from MOG35-55-treated M2-Mϕ.

## 4. Discussion

The study of macrophage polarization and their subsequent characterization and nomenclature is no easy task. It was defined a “tower of Babel” in immunology mainly due to their high plasticity, lack of exclusive or unequivocal markers that reflect the absence of a “black or white” phenotype, and inconsistent in vitro and in vivo experimental settings. This has led many experts on macrophages to come up with novel experimental guidelines and a framework for M1 and M2 populations, which necessarily requires a multiparametric profile that extensively characterizes these cells in terms of expression of several surface markers, cytokine/chemokine production, and metabolic features [9]. Based on such new guidelines, not only did we substantiate the expression profiling of macrophage differentiation in the presence of M-CSF and subsequent polarized activation into M1 or M2 cells, but we also provided novel *bona fide* markers for M2 polarization including CD11c, CD44, and CD115. To the best of our knowledge, we performed the first complete characterization of toll-like receptors in both macrophage populations. More importantly, the present study was designed to investigate possible alterations in macrophages plasticity that might be involved in the pathogenesis of multiple sclerosis by applying such large-scale characterization of M1 and M2 cells obtained from the animal model of MS. Upon induction of EAE, the whole functional profile of both macrophage populations undergoes a major alteration, characterized by an hyperactivated and even more pro-inflammatory profile for M1 and a switch of anti-inflammatory M2 to pro-inflammatory M1 macrophages. Indeed, the observed upregulation of several M1-associated markers (iNOS, CD40, CD80) and pro-inflammatory cytokines and chemokines account for stronger macrophage-dependent immune responses during the disease. This is particularly important because such markers are strictly associated with the induction of T-cell activation [18,19,20], as well as the generation of highly pathogenic TH1 and TH17 cells [21,22] that critically participate in MS immunopathogenesis. The enhanced release of key chemokines, such as CCL2 by M1-Mϕ during EAE, is notable because they are strongly associated with the onset and severity of demyelination [23,24], as well as the recruitment of further M1-Mϕ in the brain [25]. Thus, this sustains a proinflammatory self-amplifying loop, which ultimately determines MS-associated neuroinflammation and neurodegeneration. More interestingly, EAE-induced macrophage polarization to M2 was associated with a dramatic loss of their immunoregulatory phenotype, due to a major downregulation of several signature M2 markers including phagocytosis-associated CD206. CD44, and CCL22, all of which endow them with the ability to recruit TH2 and regulatory T cells [26,27]. They can also acquire M1 properties, including the expression of iNOS, CD40, and CD80/86. This agrees with previous data showing an M2-to-M1 switch in several neurodegenerative diseases, including a study on Theiler’s murine encephalomyelitis, the infectious model for chronic-progressive multiple sclerosis, whereby the onset of virus-induced demyelination was associated with a dominating M1 polarization and a loss of M2 function [14,28]. Moreover, direct administration of M2 macrophages, or pharmacological treatments that skewed M1/M2 balance towards an M2 phenotype, are able to ameliorate symptoms in these animals [14,29,30]. These findings suggest that during EAE, peripheral monocyte-derived M2-Mϕ, even in the presence of M2-polarizing conditions, switch to the M1 phenotype. This hypothesis was confirmed by our data, which we obtained by immunizing control monocyte-derived M1-Mϕ and M2-Mϕ with the highly encephalitogenic peptide myelin oligodendrocyte glycoprotein (MOG35-55), by which M1 and M2 phenotypes partially mimicked those obtained from EAE-induced macrophages, possibly indicating that the changes in macrophage function could play an important role in the pathogenic mechanisms triggered by this peptide upon the induction of encephalomyelitis.

Our work also reports for the first time a detailed TLR expression profile in M1-Mϕ and M2-Mϕ in both CFA and EAE mice. TLRs have been proposed to critically impact the onset and severity of MS [31,32,33]. Our data show that M1 and M2 populations feature quite distinct TLR transcriptional fingerprints that undergo severe reconfiguration upon the induction of EAE, with the former downregulating TLR1, 5, 6, and 8, while the latter massively upregulating TLR2. These receptors might play very different role in the disease. In fact, TLR4-/- mice are more susceptible to EAE [32], which suggests an immunomodulatory role for these receptors. Accordingly, we were able to observe a considerable downregulation of TLR6 gene in both M1- and M2-Mϕ during EAE, even though TLR4 expression remained unchanged in both subtypes, and represented the most highly expressed TLR in M2 subset. Of note, in EAE mice, M1 expressed high levels of TLR2 mRNA, while M2 considerably upregulated the TLR2 gene. This is in line with recent reports that proposed a pathogenic role of this receptor in MS [34,35]. Remaining TLRs (i.e., TLR3, 7 and 9) showed no variation between CFA and EAE mice, even though TLR7 maintained high levels of expression in both M1- and M2-Mϕs, which could explain the ameliorative effects, reported elsewhere, exerted by its specific activation on EAE clinical outcome [36].

## 5. Conclusions

**Figure 8:**
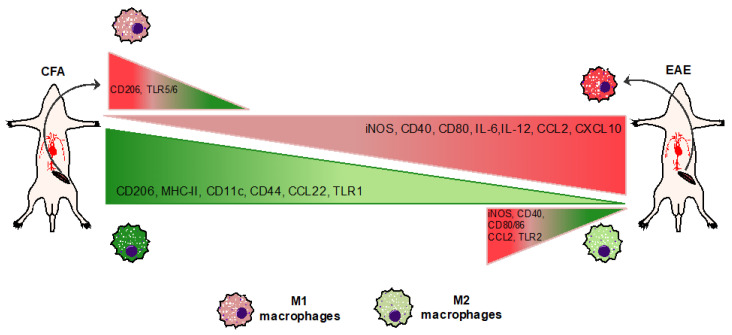
**Summary of M1/M2-Mϕ immunophenotype in CFA and EAE mice.** Induction of EAE delineates an ex vivo immunophenotypic alteration of M1/M2-Mϕ, with M1 being hyperactive and more pro-inflammatory while M2 being less protective.

In conclusion, this study reports, for the first time, a complete immunophenotypical characterization of M1 and M2 macrophages in an experimental model of MS. It also delineates the existence of a disrupted balance between these two cell types (as summarized in Figure 8) during the disease, with the former being M1 (hyperactive and more pro-inflammatory) and M2 (less protective and switching to an M1-like phenotype). These findings, although yet to be associated to further mechanistic insights, might shed light on novel pathogenic macrophage-driven mechanisms underlying MS, which can be eventually targeted with the aim of establishing an anti-inflammatory and neuroprotective M2-like phenotype.

## Figures and Tables

**Table 1:** Antibodies used for the immunophenotypic characterization of M1 and M2 macrophages obtained from CFA and EAE mice.

Antibody	Manufacturer	Dilution
CD11b APC-Cy7	BD Pharmingen	1:80
CD11c APC	BD Pharmingen	1:80
CD25 APC	BD Pharmingen	1:80
CD40 PE	Miltenyi Biotec	1:50
CD44 APC	eBioscience	1:50
CD62L FITC	BD Pharmingen	1:50
CD68 Cy5	Milenyi Biotec	1:100
CD80 FITC	Miltenyi Biotec	1:50
CD86 PE	Miltenyi Biotec	1:50
CD115 PE	Miltenyi Biotec	1:50
CD206 Brilliant Violet	Biolegend	1:100
MHC-II APC	Miltenyi Biotec	1:100
CCR5 PE	BD Pharmingen	1:50
F4/80 PerCP5.5	Biolegend	1:100

## Data Availability

Not applicable.

## Supplementary Materials

The following are available online at https://www.mdpi.com/article/10.3390/biom11060837/s1, Figure S1: Immunophenotype of macrophages obtained from CFA and EAE mice. Figure S2: Immunophenotype of MOG-immunized M1 and M2 macrophages obtained from CFA mice.
