# Supplementary material for: Macrophage Plasticity and Polarization Are Altered in the Experimental Model of Multiple Sclerosis
Source: Biomolecules. 2021 Jun 4;11(6):837. doi: 10.3390/biom11060837 (PMC8229971; doi:10.3390/biom11060837)
Supplement: Supplementary file 1 [file biomolecules-11-00837-s001.zip › biomolecules-1179797-supplementary.pdf]

Supplementary Figure 1

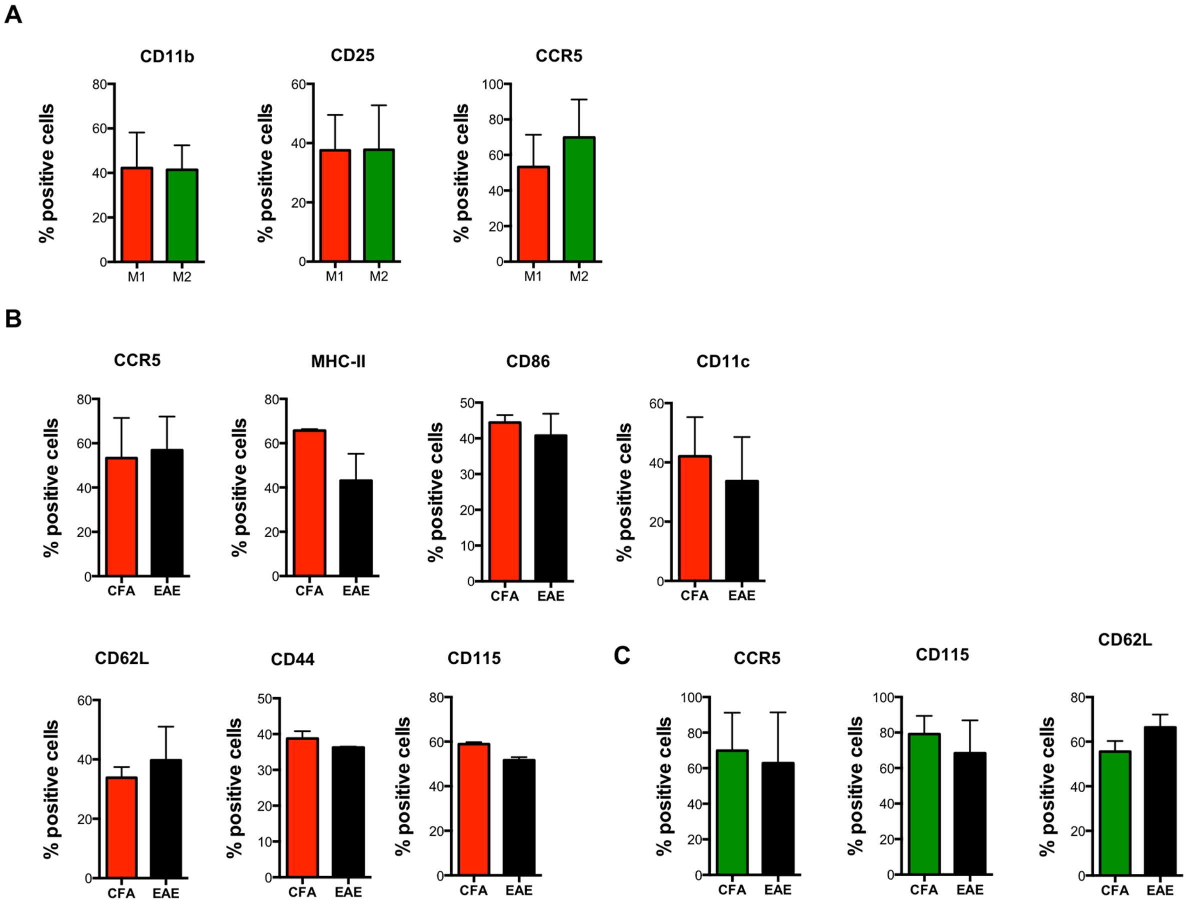

Supplementary Fig.1. Immunophenotype of macrophages obtained from CFA and EAE mice. (A) Expression of CD11b, CD25 and CCR5 by flow cytometry in M1 and M2 macrophages obtained from CFA mice. (B) Expression of CCR5, MHC-II, CD86, CD11c, CD62L, CD44 and CD115 by flow cytometry in M1-Mϕ obtained from CFA mice. (C) Expression of CCR5 and CD115 by flow cytometry in M2-Mϕ obtained from CFA mice. Data are reported as percentage of positive cells and are representative of eight independent experiments ± sem.

Supplementary Figure 2

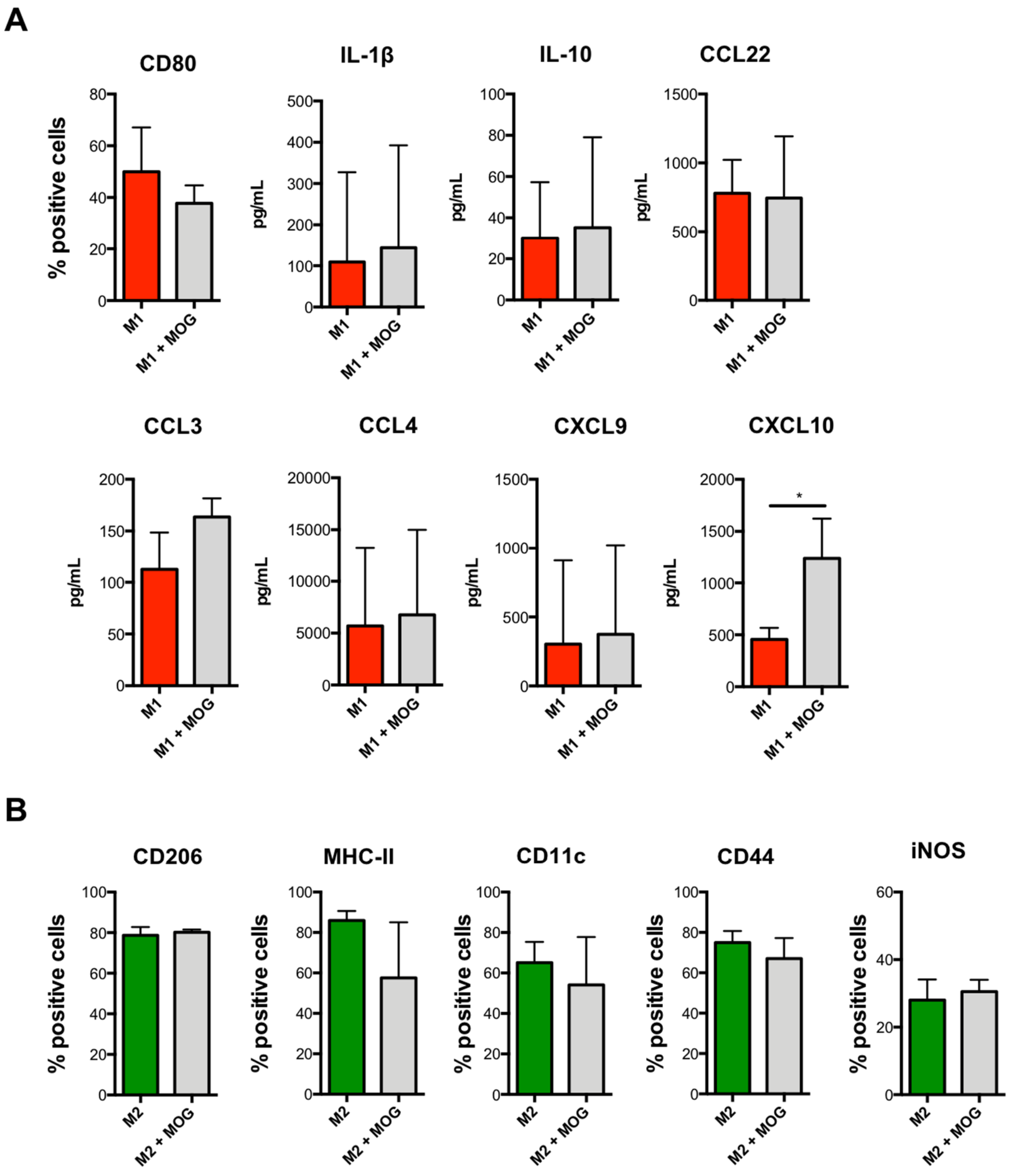

Supplementary Fig.2. Immunophenotype of MOG-immunized M1 and M2 macrophages obtained from CFA mice. (A) Expression of CD80 by flow cytometry and ELISA of IL-1 β, IL-10, CCL22, CCL3, CCL4, CXCL9 and CXCL10 in MOG-immunized M1-Mϕ obtained from CFA mice. (B) Expression of CD206, MHC-II, CD11c, CD44 and iNOS by flow cytometry in MOG-immunized M2-Mϕ obtained from CFA mice. Data are reported as percentage of positive cells and are representative of eight independent experiments ± sem.
